# Synergistic Antimicrobial Effects of Baicalin Combined with Kanamycin Against MRSA: Underlying Mechanisms and Diminished Colonization on Lettuce

**DOI:** 10.3390/ph18101458

**Published:** 2025-09-28

**Authors:** Xin Meng, Zhiyun Yu, Chao Ning, Mingtong Sun, Mengna Kang, Haiyong Guo

**Affiliations:** College of Life Science, Jilin Normal University, Siping 136000, China; 18624395651@163.com (X.M.); yuzhiyun0808@163.com (Z.Y.); 18643477064@163.com (C.N.); sunmingtong2024@163.com (M.S.); kang000719@163.com (M.K.)

**Keywords:** baicalin, kanamycin, MRSA, biofilm, antimicrobial mechanism, lettuce, combination treatment, foodborne pathogen control

## Abstract

**Background:** The treatment of methicillin-resistant *Staphylococcus aureus* (MRSA) infections is extremely challenging due to its antibiotic resistance, and the combination of plant active ingredients with antibiotics represents a potential strategy to address this issue. **Methods:** We determined the combinatorial relationship between baicalin (BA) and kanamycin (KM) using the checkerboard dilution method. The antibacterial activity of the baicalin–kanamycin (BA/KM) combination was evaluated through growth curve determination assays and scanning electron microscopy (SEM). The effects of the BA/KM combination on the cell membrane and cell wall of MRSA were analyzed using reactive oxygen species (ROS) detection assays, intracellular protein leakage experiments, alkaline phosphatase (AKP) activity assays, laser scanning confocal microscopy (LSCM) observations, and molecular docking simulations. The antibiofilm activity and related mechanisms of the BA/KM combination were elucidated via crystal violet staining, MTT assay, phenol-sulfuric acid method, congo red staining, staphyloxanthin determination assays, and quantitative real-time polymerase chain reaction (qPCR). The safety of the BA/KM combination was assessed through hemolytic activity analysis, and its anti-MRSA efficacy was evaluated on lettuce. **Results:** BA/KM combination showed a synergistic antibacterial effect on MRSA USA300. Mechanistic studies revealed that BA may interact with amino acid residues of peptidoglycan synthetase PBP2a to hinder peptidoglycan synthesis, thereby facilitating KM penetration through the cell wall. Subsequently, BA binds to amino acid residues of the membrane transporter NorA, leading to disruption of cell membrane homeostasis and enhancing KM’s ability to induce intracellular ROS accumulation in MRSA. Furthermore, the BA/KM combination reduced MRSA biofilm formation by 77.85% and decreased the metabolic activity of biofilm cells by 42.93% through inhibiting the synthesis of biofilm components EPS and PIA. Additionally, this combination suppressed the synthesis of staphyloxanthin and downregulated the expression of *agrA* and *agrC* genes. When 1/8 MIC BA was combined with 1/4 MIC KM, the count of MRSA on lettuce surfaces was reduced by 0.88 log CFU/cm^2^, an effect comparable to that of 0.2% (*v*/*v*) hydrogen peroxide. **Conclusions:** According to these findings, the BA/KM combination may offer a promising option for enhancing antibacterial efficacy through synergism, reducing antibiotic usage concentrations, and limiting MRSA transmission in fresh agricultural products.

## 1. Introduction

With the improvement of living standards, food safety has increasingly attracted public attention. Food spoilage caused by bacterial proliferation not only results in food deterioration but also possesses the potential to induce foodborne diseases, thereby posing a potential threat to human health [1]. Among various pathogenic microorganisms, methicillin-resistant *Staphylococcus aureus* (MRSA)—a Gram-positive bacterium resistant to antibiotics such as methicillin and oxacillin—emerges as a major culprit underlying foodborne illnesses [2]. The biofilm formed by MRSA represents a central factor contributing to its enhanced drug resistance and persistent infections. Bacteria encapsulated within biofilms exhibit 10–1000 times greater resistance to antibiotics and host defense mechanisms compared to free-floating planktonic bacteria, which undoubtedly elevates the challenges associated with clinical treatment and food safety management [3].

Notably, the synergistic interaction between plant-derived bioactive substances and antibiotics has been proven effective in combating drug-resistant bacteria [4]. Baicalin (BA), a flavonoid compound extracted from the plant *Scutellaria baicalensis*, has been extensively investigated for its antimicrobial and anti-biofilm properties, including interference with bacterial quorum sensing (QS) systems, disruption of biofilm architectures, and inhibition of bacterial cell membrane integrity [5,6]. Kanamycin (KM), an aminoglycoside antibiotic, is commonly utilized for the treatment of infections caused by Gram-positive and Gram-negative bacteria [7,8]. However, excessive usage may lead to ototoxicity, nephrotoxicity, and the development of bacterial resistance [9,10]. Thus, the combined application of BA and KM is anticipated to enhance inhibitory efficacy against MRSA while reducing KM dosage. Nevertheless, the synergistic antimicrobial mechanism of BA in combination with KM against MRSA remains poorly elucidated. Against this backdrop, this study aims to explore the antimicrobial activity and underlying mechanisms of their combined use against MRSA, as well as evaluate their inhibitory effect on MRSA contamination on lettuce surfaces. It is anticipated that this research will provide theoretical support and practical references for curbing foodborne MRSA infections.

## 2. Results

### 2.1. Determination of MIC

Table 1 provides a summary of the antibacterial effects exhibited by BA and KM individually and in combination against MRSA USA300 and ATCC29213. The findings revealed that the MIC values of BA against MRSA USA300 and ATCC29213 were 1250 μg/mL and 2500 μg/mL, respectively, while the MICs of KM against these two strains were 7.5 μg/mL and 1.875 μg/mL. Additionally, the MICs of GM, which served as the positive control, against MRSA USA300 and ATCC29213 were 1.56 μg/mL and 0.78 μg/mL in turn. When KM was combined with 1/8 MIC of BA, its antibacterial activity was strengthened. Specifically, the MIC against MRSA USA300 was decreased by 4-fold, indicating a synergistic effect, and the MIC against ATCC29213 was reduced by 2-fold, showing a partial synergistic effect.

### 2.2. Effect of Baicalin/Kanamycin (BA/KM) Combination on Bacterial Growth and Morphology

**Figure 1:**
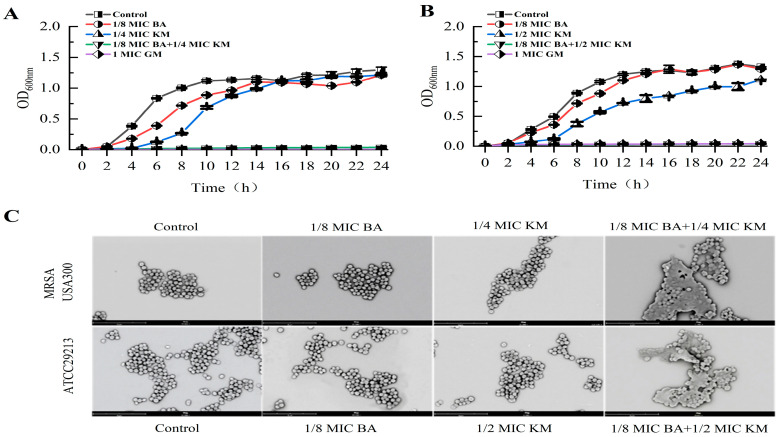
Growth inhibition of (**A**) MRSA USA300 and (**B**) *Staphylococcus aureus* ATCC29213 following treatment with BA and KM; (**C**) scanning electron micrographs of MRSA USA300 and *Staphylococcus aureus* ATCC29213 cells, each treated with different drugs (1/8 MIC BA, 1/4 MIC or 1/2 MIC KM, and the combination of the two drugs). Error bars represent standard deviations (n = 3).

When 1/8 MIC BA was combined with 1/4 MIC or 1/2 MIC KM, both bacterial strains were fully inhibited within 24 h (Figure 1A,B). In contrast, the inhibitory effect of BA or KM used alone on these two bacteria did not exceed 4 h (Figure 1A,B). These findings demonstrate that the BA/KM combination can achieve complete inhibition of MRSA USA300 and ATCC29213 growth within 24 h. Subsequently, scanning electron microscopy (SEM) observations revealed that when MRSA USA300 and ATCC29213 were treated with BA alone at 1/8 MIC, or with KM alone at 1/4 MIC (for MRSA USA300) and 1/2 MIC (for ATCC29213), respectively, the bacterial cell morphology remained consistent with that of the untreated group, exhibiting an intact structure, smooth surface, and clear edges (Figure 1C). On the other hand, after combined treatment with 1/8 MIC BA and KM (1/4 MIC or 1/2 MIC), both bacterial strains exhibited cellular breakdown, contraction, and leakage of internal substances. These findings demonstrate that the combined application of BA and KM increases damage to bacterial cell membranes, ultimately resulting in cell death.

### 2.3. Effect of BA/KM Combination on Bacterial Cell Membrane Permeability

**Figure 2:**
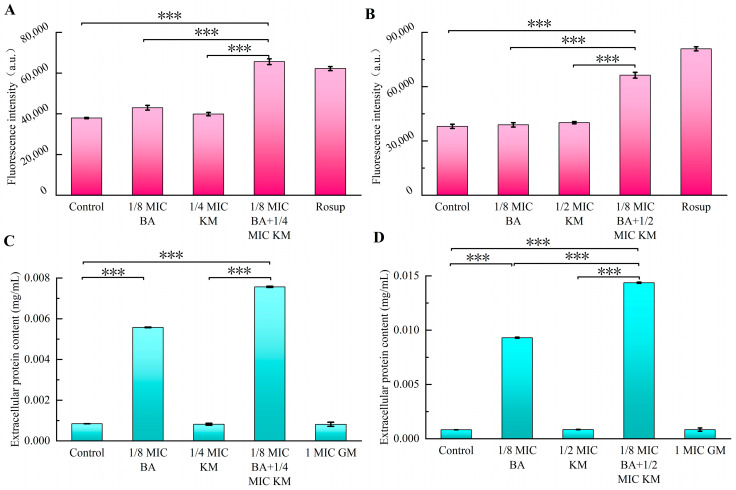
Effect of combined BA and KM treatment on the cell membrane permeability of *Staphylococcus aureus*: (**A**,**B**) intracellular reactive oxygen species (ROS) levels in MRSA USA300 and *Staphylococcus aureus* ATCC29213; (**C**,**D**) extracellular protein contents in MRSA USA300 and *Staphylococcus aureus* ATCC29213. Error bars represent standard deviations (n = 3). *** *p* ≤ 0.001.

Changes in bacterial cell membrane permeability can facilitate the buildup of intracellular ROS and the leakage of intracellular proteins, worsening bacterial functional impairments [11,12]. To explore how the BA/KM combination affects bacterial cell membrane permeability, ROS detection experiments and intracellular protein leakage assays were performed. In the ROS detection assay, untreated MRSA USA300 and ATCC29213 showed weak fluorescence intensity, indicating a low basal level of intracellular ROS under normal conditions (Figure 2A,B). When treated with BA or KM alone, the fluorescence intensity of both bacterial strains remained nearly unchanged compared to the control group. When the two bacterial strains were treated with the BA/KM combination, the fluorescence intensity was significantly increased compared with that in the groups treated with BA alone or KM alone (Figure 2A,B). These results revealed that the BA/KM combination could significantly boost the accumulation of intracellular ROS in MRSA USA300 and ATCC29213, with a promoting effect distinctly stronger than that of the single-agent treatment groups. Subsequently, a BCA protein assay kit was employed to assess the impact of the BA/KM combination on the leakage of intracellular bacterial proteins. As illustrated in Figure 2C,D, the BA/KM combination significantly increased the release of intracellular bacterial proteins compared with the control group and the KM alone group. Notably, compared with the BA alone group, the BA/KM combination significantly increased the intracellular protein leakage of *Staphylococcus aureus* ATCC 29213, while it showed no significant difference in the intracellular protein leakage of MRSA USA300. Additionally, the amount of bacterial cell protein leakage induced by BA alone was significantly greater than that induced by KM alone. Taken together, these findings demonstrate that BA plays a primary role in disrupting bacterial cell membranes and increasing membrane permeability when combined with KM.

### 2.4. Effect of BA/KM Combination on Bacterial Cell Wall Permeability

**Figure 3:**
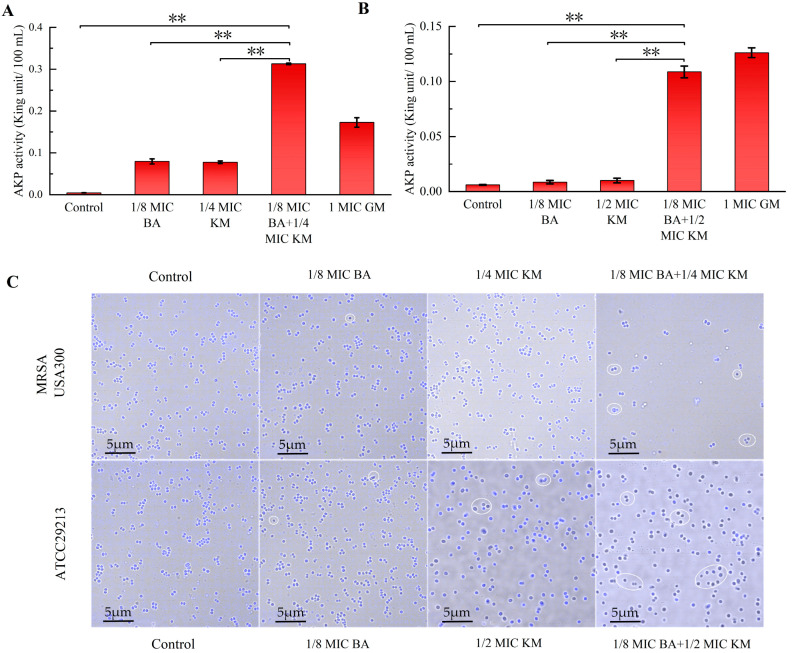
Effect of combined BA and KM treatment on the cell wall permeability of *Staphylococcus aureus*: (**A**,**B**) alkaline phosphatase (AKP) activity in MRSA USA300 and *Staphylococcus aureus* ATCC29213 following combined treatment; (**C**) peptidoglycan synthesis in MRSA USA300 and *Staphylococcus aureus* ATCC29213, observed via confocal laser scanning microscopy (CLSM). Scale bar = 5 μm. The regions of interest are marked by white circles, where a decrease in fluorescence intensity and abnormal morphology of some cells can be observed. Error bars represent standard deviations (n = 3). ** *p* ≤ 0.01.

As shown in Figure 3A, both individual and combined use of BA and KM led to an increase in extracellular AKP levels in MRSA USA300. In particular, when BA was combined with KM, the extracellular AKP activity was 78-fold and 4-fold higher than that of the untreated group and the 1/4 MIC KM alone group, respectively. For the ATCC29213 strain, neither BA alone nor KM alone effectively promoted AKP release. However, after combined treatment, AKP activity increased significantly, being 18-fold and 11-fold higher than that of the untreated group and the 1/4 MIC KM alone group, respectively (Figure 3B). Therefore, BA can enhance the permeability of KM to the cell walls of these two bacterial strains, promoting AKP release, with a more significant synergistic effect on MRSA strains. Subsequently, the HADA in situ labeling method was used to investigate the impact of the BA/KM combination on bacterial peptidoglycan biosynthesis. As shown in Figure 3C, after combined treatment with BA and KM, the fluorescence intensity of both bacterial cells was significantly weakened, and some unlabeled cells appeared. In addition, abnormal morphologies such as increased bacterial volume and collapsed cell wall structure were observed, further confirming that the BA/KM combination inhibits peptidoglycan synthesis, disrupts cell integrity, and ultimately leads to bacterial death.

### 2.5. Molecular Docking Studies on the Interactions Between BA and Peptidoglycan Synthases/Membrane Transporters

**Figure 4:**
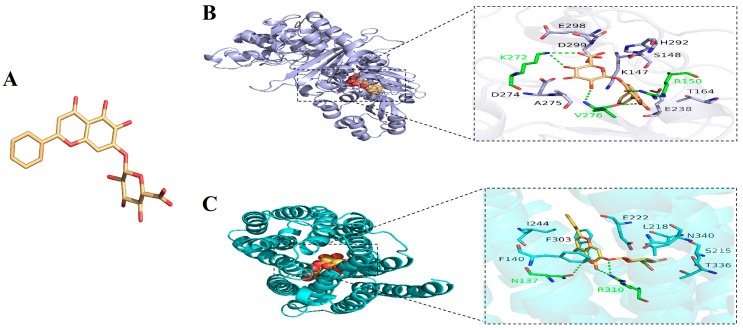
(**A**) Three-dimensional structure of BA; overview of the top-ranked docking conformations of BA with (**B**) penicillin-binding protein 2a (PBP2a) and (**C**) NorA efflux pump.

This study further explored the binding mode and potential binding sites between BA and PBP2a through molecular docking. Figure 4A shows the three-dimensional structure of BA, while Figure 4B presents the optimal docking models of BA with PBP2a. The results demonstrated that BA can specifically embed into the active cavity of PBP2a, and this binding site forms a three-dimensional interaction network composed of 12 amino acid residues, including Lys272, Asp299, Ser148, His292, Arg150, Glu298, Val276, Ala275, Thr164, Glu238, Lys147, and Asp274 (Figure 4B). In this structure, the polar groups of BA form a network of 5 sets of hydrogen bonds with Lys272, Arg150, and Val276, and its hydrophobic regions attract and tightly bind to 9 surrounding amino acids (such as Ala275, Thr164, etc.) (Figure 4B). This multi-level interaction mode endows BA with a high binding affinity for PBP2a. The prediction results from AutoDock software showed that the binding energy between BA and PBP2a was −8.830 kcal/mol. Furthermore, we found that BA can increase cell membrane permeability, thereby enhancing the antibacterial activity of KM (Figure 2). As a membrane transporter protein of *Staphylococcus aureus*, NorA is closely associated with cell membrane permeability [13]. Thus, this study also investigated the binding mode and potential binding sites between BA and NorA. AutoDock software predictions revealed that the binding energy between BA and NorA was −9.207 kcal/mol. Figure 4C displays the optimal docking models of BA with NorA. The results indicated that BA could stably bind to the cavity of NorA, which is jointly formed by amino acid residues including Arg310, Leu218, Ser215, Thr336, Glu222, Asn340, Ile244, Phe303, Asn137, and Phe140. Among these, BA forms 3 sets of hydrogen bonds with Arg310 and Asn137 around the pocket and generates hydrophobic interactions with 8 surrounding amino acids (Figure 4C). These two types of interactions synergistically enhance binding stability and further consolidate the binding effect. It can thus be concluded that the effective binding of BA to the active sites of PBP2a and NorA may be the potential molecular mechanism underlying its influence on the integrity of cell walls and cell membranes.

### 2.6. Anti-Biofilm Activity of BA/KM Combination Against Bacteria

**Figure 5:**
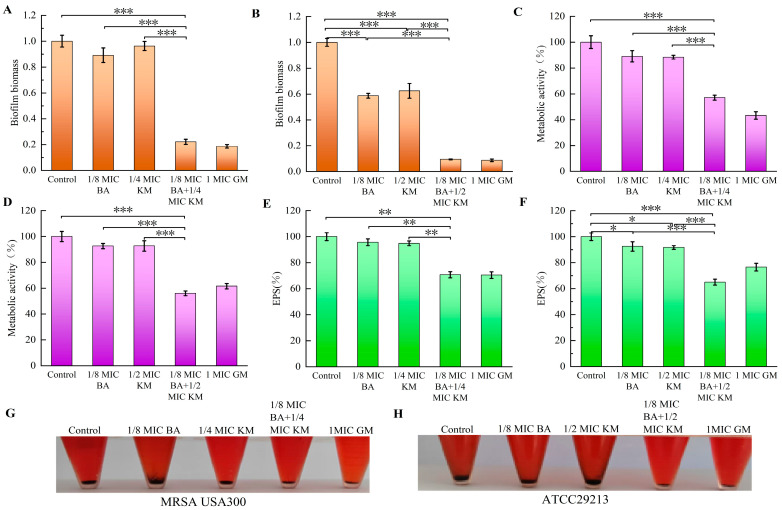
Antibiofilm activity of the BA/KM combination against *Staphylococcus aureus*. Effect of combined treatment on (**A**,**B**) biofilm formation, (**C**,**D**) biofilm metabolic activity, (**E**,**F**) extracellular polysaccharide (EPS) content, and (**G**,**H**) polysaccharide intercellular adhesin (PIA) synthesis in MRSA USA300 and *Staphylococcus aureus* ATCC29213. Error bars represent standard deviations (n = 3). * *p* ≤ 0.05, ** *p* ≤ 0.01, *** *p* ≤ 0.001.

The anti-biofilm activity of the BA and KM combination was assessed using the crystal violet staining method. The results indicated that for the MRSA USA300 strain, the inhibition rate of KM alone on biofilm formation was merely 3.68%. In contrast, when combined with BA, KM’s inhibitory activity was significantly strengthened, with the inhibition rate reaching 77.85% (Figure 5A). For the ATCC29213 strain, compared with the KM monotherapy group (which had an inhibition rate of 37.52%), the inhibitory effect on biofilms after the BA/KM combination treatment was significantly improved, with an increase of 53.06% (Figure 5B).

### 2.7. Effect of BA/KM Combination on Metabolic Activity of Bacterial Biofilm Cells

The conversion of MTT to formazan directly depends on cellular respiratory activity and is positively correlated with cell viability. Therefore, in this study, the MTT assay was used to determine the number of metabolically active cells in bacterial biofilms following combined treatment with BA and KM [14]. As shown in Figure 5C,D, compared with the control group, the metabolic activity of biofilm cells of MRSA USA300 and ATCC29213 decreased by approximately 10% when treated with BA or KM alone. In contrast, when BA was combined with KM, the metabolic activity of biofilm cells of these two strains decreased by 42.93% and 43.98%, respectively. This significant reduction in metabolic activity indicates that the viability of both bacterial strains is relatively low under the combined treatment of BA and KM.

### 2.8. Effects of BA/KM Combination on the Formation of Biofilm Components: EPS and PIA

Extracellular polymeric substances (EPS), serving as the core component of bacterial biofilms, make up over 90% of the biofilm dry weight [15]. Through various interactions with bacterial aggregates, EPS sustain the structural stability, metabolic activity, and microecological equilibrium of biofilms [15]. They are also key factors that allow biofilms to withstand external pressures and achieve persistent colonization [15]. Therefore, dispersing or degrading EPS can weaken the ability of biofilm formation, providing an important intervention target for the control of related infections. The results of this study showed that compared with the control group and the single-drug treatment groups, the BA/KM combination significantly reduced the EPS content in MRSA USA300 and ATCC29213 strains (Figure 5E,F), with reduction rates of 29.27% and 34.98%, respectively. This indicates that the BA/KM combination can significantly reduce the synthesis of EPS in the biofilms of *Staphylococcus aureus* (MRSA USA300 and ATCC29213 strains), thereby enhancing the clearance of bacteria within the biofilms.

The main component of EPS in *Staphylococcus* sp. is polysaccharide intercellular adhesin (PIA), which is synthesized by proteins encoded by the *ica* operon (including *icaA*/*icaD* genes and other genes) [16]. PIA mediates bacterial surface adhesion and slime production, and serves as a core substance for bacterial biofilm formation and enhanced resistance to environmental stresses. Additionally, it can bind to Congo red, resulting in the formation of black precipitates after centrifugation [17]. Lee et al. cultured bacteria using the Congo red broth method and further determined whether strains could synthesize PIA by observing color changes (turning black) of the broth [18]. The results showed that all 37 *icaA*/*icaD*-positive strains (with PIA synthesis capability) turned the TSB-based Congo red broth black, while none of the 10 *icaA*/*icaD*-negative strains (without PIA synthesis capability) could induce such a color change [18]. Therefore, we used this method to evaluate the inhibitory effect of the BA/KM combination on bacterial PIA synthesis. As shown in Figure 5G,H, the control group had the highest amount of black precipitate, indicating the highest PIA synthesis. Compared with the single KM treatment, the amount of black precipitate in MRSA USA300 and ATCC29213 strains was reduced after the combined treatment with BA and KM. This suggests that the combined treatment can more efficiently inhibit the synthesis of PIA, a key component of biofilms, thereby disrupting the structural stability of biofilms and promoting bacterial death.

### 2.9. Effects of BA/KM Combination on Bacterial Virulence Factors

**Figure 6:**
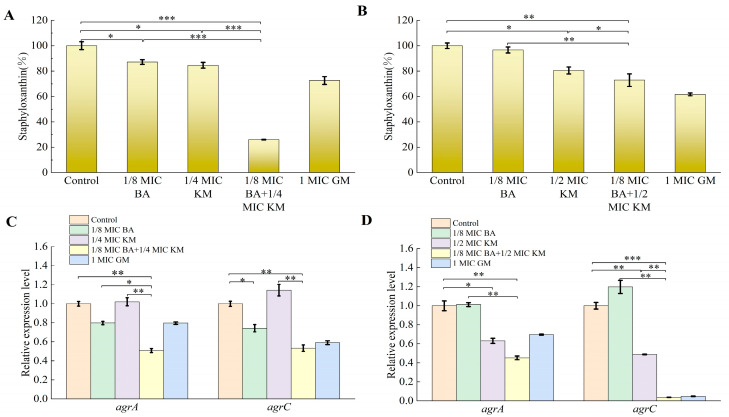
Effect of combined BA and KM treatment on virulence factor synthesis and quorum-sensing gene expression in *Staphylococcus aureus*: (**A**,**B**) staphyloxanthin content in MRSA USA300 and *Staphylococcus aureus* ATCC29213; (**C**,**D**) relative expression levels of the *agrA* and *agrC* quorum-sensing genes in MRSA USA300 and *Staphylococcus aureus* ATCC29213. Error bars represent standard deviations (n = 3). * *p* ≤ 0.05, ** *p* ≤ 0.01, *** *p* ≤ 0.001.

Virulence factors secreted by *Staphylococcus aureus* play a key role in biofilm formation and the evasion of the host’s innate immune defense [19]. Staphyloxanthin, a carotenoid pigment synthesized by this bacterium, not only shields *Staphylococcus aureus* from phagocytosis but also strengthens the stability of the bacterial cell membrane by organizing the alkyl chains of membrane lipids, thereby extending the bacterium’s survival duration [20]. This experiment aimed to evaluate the effect of the BA/KM combination on staphyloxanthin synthesis in *Staphylococcus aureus*. As shown in Figure 6A, for the MRSA USA300 strain, compared with the control group, treatment with BA or KM alone reduced staphyloxanthin synthesis by 12.85% and 15.41%, respectively, whereas the combined use of BA and KM significantly enhanced the inhibitory effect on staphyloxanthin formation in this strain, with a reduction of 74%. For the ATCC strain, compared with the control group, KM alone reduced staphyloxanthin synthesis by 19.5%. The inhibitory effect was further enhanced when BA was combined with KM, resulting in a reduction of 27.1% (Figure 6B). These results indicate that the BA/KM combination can effectively reduce the release of virulence factors in *Staphylococcus aureus*.

### 2.10. Effects of BA/KM Combination on the Expression of Bacterial Quorum-Sensing Genes

As core components of the *agr* system in *Staphylococcus aureus*, *agrA* and *agrC* are closely associated with biofilm, metabolism, and virulence factors through a complex regulatory network [21]. Therefore, this study evaluated the effect of the BA/KM combination on the expression of quorum-sensing genes *agrA* and *agrC* in bacteria. As shown in Figure 6C, compared with the control group, both BA alone and the BA/KM combination downregulated the expression of *agrA* and *agrC* genes to varying degrees in MRSA USA300. Specifically, BA at 1/8 MIC inhibited the expression of *agrA* and *agrC* by 20% and 26%, respectively. When the two were combined, the inhibition rates of *agrA* and *agrC* gene expression were 49% and 47%, respectively. For the ATCC29213 strain, as shown in Figure 6D, KM at 1/2 MIC inhibited the expression of *agrA* and *agrC* by 37% and 51%, respectively. The BA/KM combination resulted in more significant downregulation of *agrA* and *agrC* gene expression, with inhibition rates of 55% and 98%, respectively.

### 2.11. Safety Analysis

**Figure 7:**
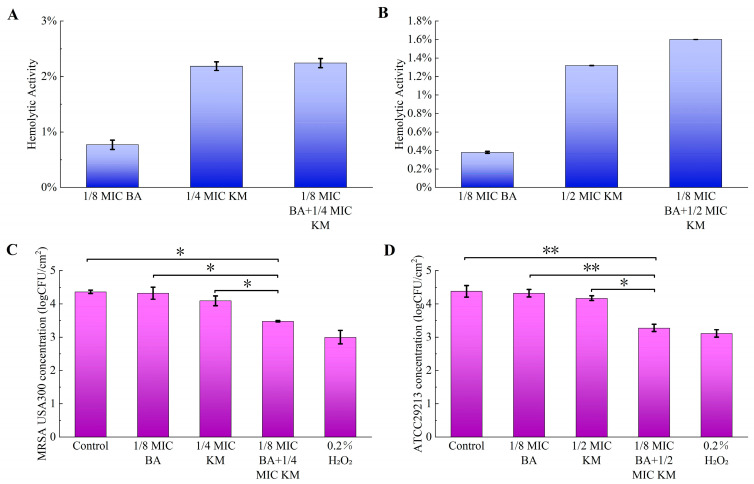
Hemolytic activity of drugs and their antibacterial efficacy in lettuce: (**A,B**) the effects of different concentrations of BA and KM on hemolytic activity against MRSA USA300 (**A**) or *Staphylococcus aureus* ATCC29213 (**B**); (**C**,**D**) antibacterial efficacy of BA and KM against MRSA USA300 (**C**) and *Staphylococcus aureus* ATCC29213 (**D**) in lettuce after 15 min of antimicrobial exposure. Error bars represent standard deviations (n = 3). * *p* ≤ 0.05, ** *p* ≤ 0.01.

To evaluate the safety of BA combined with KM, this study performed hemolytic activity assays on all drug-treated groups acting on MRSA USA300 and ATCC29213 to investigate the hemolytic effect of the drugs on red blood cells. The results showed that the hemolytic activity of all drug-treated groups was lower than 3%, indicating that within the experimental concentration range, these substances did not cause significant red blood cell rupture or hemoglobin release, exerted extremely low direct damage to red blood cells, and thus exhibited good biological safety (Figure 7A,B).

### 2.12. Efficacy of BA/KM Combination in Lettuce Model

This study evaluated the antibacterial activity of the BA/KM combination against MRSA USA300 and ATCC29213 in lettuce. As shown in Figure 7C, individual treatments with BA or KM exhibited negligible effects on the reduction in MRSA USA300, with the colony count in lettuce decreasing by a range of 0.04–0.27 log CFU/cm^2^. However, the BA/KM combination significantly reduced MRSA USA300 in lettuce by 0.88 log CFU/cm^2^. Similarly, individual treatments with BA or KM had minimal effects on the reduction in ATCC29213, with the colony count in lettuce decreasing by a range of 0.06–0.21 log CFU/cm^2^. The BA/KM combination led to a significant reduction in ATCC29213 in lettuce by 1.1 log CFU/cm^2^ (Figure 7D). Additionally, the reduction effects of the BA/KM combination on MRSA USA300 and ATCC29213 in lettuce were comparable to those of 0.2% (*v*/*v*) hydrogen peroxide (Figure 7C,D).

## 3. Discussion

The widespread contamination of methicillin-resistant *Staphylococcus aureus* (MRSA) in the food chain, along with the problem of antimicrobial resistance caused by high-concentration antibiotics in clinical treatment, has rendered the combined application strategy of plant bioactive components and antibiotics particularly important [22]. This study found that the synergistic activity of BA combined with KM against the MRSA USA300 strain (FICI of 0.375) was stronger than that against the ATCC29213 strain (FICI of 0.625) (Table 1). Compared with the use of KM alone, the BA/KM combination could completely inhibit bacterial growth within 24 h and disrupt the morphological structure of bacterial cells (Figure 1). Existing studies have clearly established that the destructive effect of BA on bacterial cell membranes is one of its core antibacterial mechanisms. Chen et al. found that BA can act on the cell membrane of *Staphylococcus aureus*, causing membrane structure disruption and increased permeability, which in turn leads to the leakage of intracellular substances [23]. The results of this study are highly consistent with the aforementioned mechanism. Through reactive oxygen species (ROS) detection experiments and intracellular protein leakage assays, it was found that BA could enhance the ability of KM to induce intracellular ROS accumulation and intracellular protein leakage in these two bacterial strains, thereby increasing the permeability of bacterial cell membranes (Figure 2). In addition, BA could enhance the permeation effect of KM on the cell walls of these two bacterial strains and promote the release of alkaline phosphatase (AKP) (Figure 3A,B), indicating that BA not only impairs the integrity of the cell membrane but also potentially interferes with cell wall synthesis. As the core component of the *Staphylococcus aureus* cell wall, peptidoglycan plays a key role in maintaining cellular structural integrity and morphological stability, leading to the speculation that it may be the core target when BA and KM act synergistically on the bacterial cell wall. Mireles et al. found that the combination of cinnamaldehyde and baicalin enhances the penetration of colistin into the cell walls of *Enterobacterales* and *Acinetobacter baumannii* by disrupting bacterial cell wall integrity and modulating cell wall charge [24]. However, the direct effect of this combination on the peptidoglycan assembly process has not yet been clarified. In this study, observations using HADA in situ labeling showed that, compared with KM alone, the BA/KM combination caused the collapse of the bacterial cell wall structure, and some cells remained unlabeled, indicating disrupted peptidoglycan synthesis. This confirms that the combined use of the two can inhibit peptidoglycan synthesis, disrupt cellular integrity, and ultimately result in bacterial death (Figure 3C). Combined with the result from molecular docking experiments that BA can bind to the active site of the cell wall protein PBP2a (Figure 4), it further confirms that BA can target PBP2a—a key protein associated with MRSA resistance. This finding provides a novel molecular explanation for BA-mediated reversal of MRSA resistance. Additionally, molecular docking experiments also revealed that BA exhibits a strong binding capacity to the cell membrane protein NorA (Figure 4), which is consistent with the conclusion of Cheng et al. that BA restores bacterial susceptibility to drugs by acting on cell membrane proteins [25]. These results may shed light on the potential molecular mechanisms by which BA impairs the integrity of bacterial cell walls and cell membranes.

The biofilm formed by *Staphylococcus aureus* can not only resist the action of antibiotics but also weaken the clearance ability of the immune system [26]. It is this dual protective mechanism that makes the infections caused by this bacterium often characterized by persistence and easy recurrence. Han et al. reported that the combination of BA and levofloxacin exerts a synergistic effect on the biofilms of high-virulence *Klebsiella pneumoniae* in vitro [27]. However, the underlying mechanism associated with this synergy has not been explored in depth. Building on the aforementioned findings, this study further expands the understanding of the antibiofilm mechanism underlying the combination of BA and antibiotics. Our results showed that the BA/KM combination can significantly inhibit the formation of bacterial biofilms and reduce the metabolic activity of biofilm cells (Figure 5A–D). Since the homeostasis of biofilms is closely related to the metabolic capacity of their constituent microorganisms [26], it can be inferred that the combined treatment with BA and KM targets and kills cells in the early stage of biofilm formation, leading to a decrease in the metabolic capacity of adherent cells and thus reducing biofilm production. Further research also found that the BA/KM combination could inhibit the biofilm component EPS, thereby enhancing the antibacterial effect on bacteria within biofilms (Figure 5E,F). This is consistent with the mechanism proposed by Addo et al. that a reduction in EPS can expose dispersed biofilm cells, making them more susceptible to inactivation by antimicrobials [28]. Moreover, this study found that the combined administration of the two could inhibit the synthesis of PIA, a key component of biofilms, thereby disrupting the structural stability of biofilms (Figure 5G,H). At the same time, the BA/KM combination could also exert anti-biofilm effects by inhibiting the synthesis of the virulence factor staphyloxanthin and downregulating the expression of the quorum-sensing genes *agrA* and *agrC* (Figure 6).

Hemolysis experiments showed that the BA/KM combination had good biological safety at the tested concentrations (Figure 7A,B). Finally, we detected the antibacterial activity of their combination against MRSA USA300 and ATCC 29213 in lettuce and found that it had a clearing effect on these two strains on the lettuce surface, reducing the number of strains by 0.88 log CFU/cm^2^ and 1.1 log CFU/cm^2^, respectively (Figure 7C,D). This result is basically consistent with the study by Jin et al., who pointed out that the combination of 1/4 MIC carvacrol and 1/2 MIC cinnamaldehyde could significantly reduce MRSA009 in lettuce by 1.4 log CFU/cm^2^ [29]. However, the combined use strategy of BA and KM is not yet applicable to conventional food processing—KM, as a clinically commonly used antibiotic, may induce antibiotic resistance in environmental microorganisms if applied on a large scale in food processing and further pose potential threats to human health through the food chain, which is contrary to the principles of rational antibiotic use and food safety regulations.

## 4. Materials and Methods

### 4.1. Bacterial Strains, Cell Cultures, and Chemicals

Methicillin-susceptible *Staphylococcus aureus* ATCC 29213 was provided by Jilin Normal University (Siping, China), and community-acquired methicillin-resistant *Staphylococcus aureus* (CA-MRSA) USA300 was kindly provided by Professor Ji from the University of Minnesota, Minneapolis, MN, USA. Both strains were aerobically cultured on tryptic soy agar (TSA, Haibo Biotechnology, Qingdao, China) at 37 °C for 24 h. Single colonies were picked and inoculated into 5 mL of tryptic soy broth (TSB, Haibo Biotechnology, Qingdao, China), followed by incubation at 37 °C for 8 h until reaching the logarithmic phase (1 × 10^8^ CFU/mL). Sterile water was used as the negative control, and 1MIC gentamicin (GM) served as the positive control in the experiment.

KM and GM were purchased from Aladdin (Shanghai, China). BA (HPLC purity ≥ 98%), Congo red (CR), crystal violet (CV), and reactive oxygen species (ROS) detection kits were obtained from Solarbio (Beijing, China). The BCA protein assay kit was purchased from BestBio (Nanjing, China). The alkaline phosphatase (AKP) assay kit was obtained from Jiancheng Bioengineering Institute (Nanjing, China). HADA (7-hydroxycoumarincarbonylamino-D-alanine) was obtained from MCE. MTT was purchased from Land Bridge Biotechnology (Beijing, China), and phenol was obtained from Yueteng Biotechnology (Shanghai, China).

### 4.2. Determination of Minimum Inhibitory Concentration (MIC)

Baicalin (BA) was first dissolved in a small amount of 5% sodium bicarbonate solution (with pH value controlled at 7.2–7.4), followed by the addition of sterile water for a two-fold dilution to achieve a final concentration ranging from 625 to 5000 µg/mL. Kanamycin (KM) and gentamicin (GM) were directly subjected to a two-fold dilution with sterile water, and the diluted concentration ranges were 0.46875–7.5 µg/mL and 0.195–3.13 µg/mL, respectively. 50 µL of the above-prepared drugs was added to each well of a 96-well plate and then mixed uniformly with 50 µL of bacterial suspension at a concentration of 2 × 10^5^ CFU/mL. After that, the 96-well plate was incubated at 37 °C for 24 h. At the end of incubation, the bacterial growth was detected at a wavelength of 600 nm using a Spark™ multifunctional microplate reader produced by Tecan (Shanghai, China) Laboratory Equipment Co., Ltd. [30].

### 4.3. Checkerboard Microbroth Assay

For both BA and KM or GM, their concentrations were initiated from their respective MICs and underwent two-fold gradient dilution along the *x*-axis and *y*-axis of the 96-well plate. After the addition of bacteria (2 × 10^5^ CFU/mL), the plates were cultured at 37 °C for 24 h. Then, the absorbance at 600 nm was measured using a Spark™ multifunctional microplate reader (Tecan (Shanghai) Laboratory Equipment Co., Shanghai, China). The fractional inhibitory concentration (FIC) index was computed using the following formula [14]: FIC index = FIC of A + FIC of B. Moreover, the FIC for each test compound was calculated based on the following formulas: FIC of A = MIC of A in combination/MIC of A when used alone, and FIC of B = MIC of B in combination/MIC of B when used alone. An FIC index of less than 0.5 signifies a synergistic effect; a range of 0.5–0.75 indicates a partial synergistic effect; 0.75–1 represents an additive effect; 1–4 denotes no effect; and greater than 4 indicates an antagonistic effect.

### 4.4. Growth Curve Assay

Bacterial suspensions (2 × 10^5^ CFU/mL) were mixed with test agents, including BA alone (1/8 MIC), KM alone (1/4 MIC or 1/2 MIC), and their combination. The mixture was cultured at 37 °C for 24 h in an automatic microbial growth curve analyzer. Monitoring was performed every 2 h, and curves were plotted with time as the *x*-axis and the difference in absorbance values as the *y*-axis to analyze the impact of drug combination on bacterial growth [31].

### 4.5. Scanning Electron Microscopy

The bacterial suspension (1 × 10^6^ CFU/mL) was combined with the test drugs, including BA alone (1/8 MIC), KM alone (1/4 MIC or 1/2 MIC), and their combination. The resulting mixture was incubated at 37 °C for 1 h. Bacterial cells were collected by centrifugation for 10 min, washed three times with phosphate-buffered saline, and fixed with 2.5% glutaraldehyde at 4 °C overnight. Following gradient dehydration using ethanol, the samples were freeze-dried and subjected to vacuum sputter coating and then observed under a scanning electron microscope [32].

### 4.6. Reactive Oxygen Species (ROS) Detection Assay

An ROS detection kit was utilized to assess the impact of the BA/KM combination on intracellular ROS levels in bacterial cells [33]. The bacterial concentration was adjusted to 1 × 10^6^ CFU/mL. The bacteria were then incubated overnight (for 24 h) with BA at 1/8 MIC, KM at 1/4 MIC or 1/2 MIC, and the mixtures of BA and KM at the aforementioned concentrations, respectively. After being washed with PBS, a 10 μM DCHF-DA probe was added, and the mixture was incubated at 37 °C in the dark for 1.5 h. Following an additional PBS wash, fluorescence intensity was determined under the parameters of an excitation wavelength of 488 nm and an emission wavelength of 525 nm. Sterile water served as the negative control, while Rosup functioned as the positive control.

### 4.7. Cell Membrane Permeability Assay

Bacterial cultures were centrifuged at 4000 rpm for 10 min, and the supernatant was discarded. After being washed twice with PBS, the bacteria were resuspended to a concentration of 1 × 10^6^ CFU/mL. Test drugs (BA at 1/8 MIC, KM at 1/4 MIC or 1/2 MIC, and the mixtures of the two drugs) were then added, and the mixture was incubated with shaking at 100 rpm at 4 °C for 6 h. The supernatant was collected by centrifugation at 4000 rpm for 10 min, and the extracellular protein content was measured using a BCA protein assay kit [34].

### 4.8. Alkaline Phosphatase (AKP) Activity Assay

In accordance with the method reported by Chen et al., bacterial suspensions were adjusted to a concentration of 1 × 10^6^ CFU/mL and exposed to BA (1/8 MIC), KM (1/4 MIC or 1/2 MIC), and their combined treatment at 37 °C for 2 h [35]. The supernatant was obtained via centrifugation at 1000× *g* for 10 min, and AKP activity was assessed using an AKP assay kit.

### 4.9. HADA Labeling

The impact of BA in combination with KM on bacterial peptidoglycan synthesis was tracked using 7-hydroxycoumarincarbonylamino-D-alanine (HADA) [36]. Bacterial suspensions with a concentration of 1 × 10^6^ CFU/mL were, respectively, incubated with BA (1/8 MIC), KM (1/4 MIC or 1/2 MIC), and their mixture at 37 °C for 4 h. After being washed and resuspended in PBS, 250 μM HADA was added, and then the mixture was incubated with shaking at 37 °C for 30 min. Following three washes, the bacterial cells were resuspended in PBS (pH 7.4) and subjected to fluorescence imaging with a confocal laser scanning microscope (CLSM, fv4000, Olympus Corporation, Tokyo, Japan) under the conditions of an excitation wavelength of 350 nm and an emission wavelength of 460 nm.

### 4.10. Molecular Docking

The 2D molecular structure of the ligand BA was downloaded from the PubChem database, and its 3D structure was constructed using Chem3D. The X-ray structures of *Staphylococcus aureus* penicillin-binding protein 2a (PBP2a, UniProt ID:A0A4P8D748) and NorA protein (UniProt ID:Q2G2M2) were retrieved from the Universal Protein Resource (UniProt) database. The structures of the ligand and receptor molecules were processed using AutoDock Tools-1.5.6 software prior to docking [37]. In this process, BA served as the ligand, while PBP2a and NorA acted as the receptors. Energy optimization was performed using the Amber14 force field [38]. Specifically, the optimization process was carried out in two steps: first, 1000 steps of optimization using the Steepest Descent Method, followed by further optimization of the structure with 5000 steps of the Conjugate Gradient Method. The final optimized structure was used as the model for subsequent analyses.

### 4.11. Biofilm Formation Inhibition Assay

A 100 μL of bacterial suspension (1 × 10^6^ CFU/mL) was separately incubated with BA at 1/8 MIC, KM at 1/4 MIC or 1/2 MIC, and the mixtures of the two drugs, respectively, at 37 °C for 24 h. After undergoing three washes with PBS, the samples were fixed in methanol for 15 min, followed by staining with 0.1% crystal violet for 5 min. After another round of washing, 95% ethanol was added, and the mixture was shaken at room temperature for 30 min. The biofilm biomass was quantified at 590 nm using a Spark™ multimode microplate reader (Tecan (Shanghai) Laboratory Equipment Co., Ltd., Shanghai, China). Calculation of the biofilm biomass was performed using the formula: OD_590_ value of the tested sample divided by the OD_590_ value of the untreated control group [30,39].

### 4.12. Biofilm Metabolic Activity Assay

The 3-[4,5-dimethylthiazol-2-yl]-2,5-diphenyltetrazolium bromide (MTT) reduction method was used to assess bacterial metabolic activity [14]. Bacterial suspensions with a concentration of 1 × 10^6^ CFU/mL were mixed in equal volumes with BA (1/8 MIC), KM (1/4 MIC or 1/2 MIC), and their combination, respectively, before being added to a 96-well plate. The plate was then incubated at 37 °C for 36 h. After removing the supernatant, each well received 20 μL of 10 mg/mL MTT and 20 μL of TSB. Following incubation at 37 °C for 3 h, the supernatant was discarded, and 200 μL of DMSO was added to each well. After standing at room temperature for 2 min, the absorbance at 570 nm was measured. The percentage of bacterial metabolic activity was computed using the formula: (OD_570_ of the experimental group/OD_570_ of the control group) × 100%.

### 4.13. Quantification Experiment of Biofilm Component EPS

Bacterial suspension (1 × 10^6^ CFU/mL) was co-incubated with BA at 1/8 MIC, KM at 1/4 MIC or 1/2 MIC, and the mixtures of the two drugs, respectively, at 37 °C with 200 rpm shaking for 36 h. The supernatant was collected after low-temperature centrifugation at 3500× *g* for 20 min. The precipitate was resuspended in 2 mL EDTA, vortexed for 15 min, and recentrifuged at 3500× *g* and 4 °C for 20 min. This supernatant was combined with the first, and 2.2 volumes of chilled absolute ethanol were added for incubation at −20 °C for 1 h. After centrifugation at 3500× *g* and 4 °C for 20 min, the precipitate was resuspended in 1 mL sterile water following supernatant removal. Then, 1 mL of 5% phenol solution and 5 mL of concentrated sulfuric acid were added. After 5 min at room temperature, absorbance at 490 nm was measured [40]. Relative EPS content was calculated as (Experimental group OD_490_/Control group OD_490_) × 100%.

### 4.14. Experiment on PIA Synthesis in Biofilm Components

Bacterial suspensions were adjusted to a concentration of 1 × 10^6^ CFU/mL, after which Congo red (CR) at a final concentration of 1 mg/mL and the test drugs (BA at 1/8 MIC, KM at 1/4 MIC or 1/2 MIC, and the mixtures of the two drugs) were added. Following incubation at 37 °C for 48 h, the synthesis of PIA was indirectly indicated by examining the quantity of maroon precipitate that formed [18].

### 4.15. Measurement of Staphyloxanthin Production

Following the method described by Lee et al. [41], bacterial suspensions were co-incubated with different drugs (BA at 1/8 MIC, KM at 1/4 MIC or 1/2 MIC, and the mixtures of the two drugs) at 37 °C with shaking at 250 r/min for 16 h. Cells were collected by centrifugation, resuspended in DMSO, and incubated at 40 °C for 20 min. After centrifugation at 10,000× *g* for 10 min, the absorbance of the supernatant was measured at 450 nm.

### 4.16. qPCR

Bacteria (1 × 10^6^ CFU/mL) were cultured with test agents (BA at 1/8 MIC, KM at 1/4 MIC or 1/2 MIC, and the mixtures of the two drugs) for 24 h. One milliliter of each sample was centrifuged at 8000 rpm for 1 min at 4 °C. The supernatant was removed, and the pellet was resuspended in lysozyme. After 10 min of incubation, 1 mL TransZol Up was added, vortexed for 1 min, and left at room temperature for 5 min. Next, 200 μL chloroform was added to the supernatant, vortexed for 1 min, incubated for 15 min, and then centrifuged at 12,000× *g* for 15 min at 4 °C. The resulting supernatant was mixed with equal-volume isopropanol, incubated for 10 min, and recentrifuged at 12,000× *g* for 15 min at 4 °C. The pellet was washed with 75% ethanol, dried, and resuspended in DEPC-treated water. RNA integrity was checked via agarose gel electrophoresis, and cDNA was synthesized using a reverse transcription kit. Primer sequences were *agrA*-F: TGCGAAGACGATCCAAAACAAAGAG, *agrA*-R: CGGATTTCACTGCCTAATTTGATACC; *agrC*-F: TTGAAGCTATCAACAACGAAATGCG, *agrC*-R: CGCAGTAATTAAGCCTTTAATTTCACGT; *16S rRNA*-F: GCTGCCCTTTGTATTGTC, *16S rRNA*-R: AGATGTTGGGTTAAGTCCC [42]. Thermal cycling conditions: initial denaturation at 95 °C for 3 min, followed by 45 cycles of 95 °C for 15 s (denaturation) and 60 °C for 45 s (annealing/extension). Relative gene expression was calculated using the 2^(−ΔΔCt)^ method. RNase-Free dH_2_O served as the negative control, and 1MIC GM as the positive control.

### 4.17. Measurement of Hemolytic Activity

Healthy rabbit plasma underwent centrifugation at 1000× *g* for 10 min to remove serum, after which it was washed three times with PBS and diluted to form a 1% blood cell suspension [43]. The test agents (BA at 1/8 MIC, KM at 1/4 MIC or 1/2 MIC, and the mixtures of the two drugs) were incubated with these blood cells at 37 °C for 1 h and then centrifuged again at 1000× *g* for 10 min. A 100 μL portion of the supernatant was collected to determine the absorbance at 570 nm. PBS was used as the negative control, while 0.2% Triton X-100 served as the positive control. The hemolysis rate was computed using the formula: [(OD of test agent − OD of PBS)/(OD of Triton X-100 − OD of PBS)] × 100%.

### 4.18. Applications on Lettuce

The experimental operation was carried out with reference to the method reported by Jin et al. [29], with specific steps as follows: Fresh lettuce leaves (2.5 × 2.5 cm in size) were prepared and sterilized using 75% ethanol. Next, the sterile lettuce slices, after being air-dried, were immersed in the bacterial solution (10^6^ CFU/mL) for 1 min [29]. The initial inoculation amounts of MRSA USA300 and ATCC29213 on the lettuce were 5.54 log CFU/cm^2^ and 5.57 log CFU/cm^2^, respectively. Later, the inoculated lettuce slices were soaked in solutions containing BA (1/8 MIC), KM (1/4 MIC or 1/2 MIC), or their combination, respectively. In the experiment, 0.2% (*v*/*v*) hydrogen peroxide was employed as the positive control, and sterile deionized water as the negative control. The diluted samples were spread onto MH agar plates and incubated at 37 °C for 24 h. Bacterial colonies were counted using the plate counting method to assess the inhibitory effect of the BA and KM combination on MRSA-contaminated lettuce.

### 4.19. Statistical Analysis

Each experiment was replicated a minimum of three times. Results are presented as the mean ± standard deviation (SD). Statistical significance was evaluated using a paired Student’s t-test, with significance levels indicated as follows: * for *p* < 0.05, ** for *p* < 0.01, and *** for *p* < 0.001.

## 5. Conclusions

This study reveals the antibacterial and anti-biofilm potential of the BA/KM combination against MRSA, and this finding is of great significance for the food safety of vegetable products. The results show that the BA/KM combination can synergistically inhibit the growth of MRSA. The underlying mechanism is as follows: By interacting with the amino acid residues of NorA protein and peptidoglycan synthetase PBP2a, BA promotes the accumulation of ROS, the leakage of intracellular proteins, and the release of AKP, while blocking peptidoglycan synthesis and disrupting the integrity of bacterial cell membranes and cell walls, ultimately significantly enhancing the antibacterial activity of KM against MRSA. In addition, the BA/KM combination exerts anti-biofilm effects through multiple pathways, including inhibiting biofilm formation, reducing the metabolic activity of biofilm cells, suppressing the synthesis of biofilm components (EPS and PIA) and virulence factors, and downregulating the expression of quorum-sensing genes *agrA* and *agrC*. Furthermore, the BA/KM combination shows good biological safety at the tested concentrations and exhibits strong inhibitory effects on MRSA in lettuce, providing critical empirical support for research on synergistic bacterial control using natural active ingredients and low-dose antibiotics. It should be noted that this study still has several limitations that require further improvement, and future research can be expanded around the following directions: First, this study was entirely designed as an in vitro experiment. In the future, in vivo experiments are needed to further evaluate the systemic toxicity, pharmacokinetic characteristics, and actual therapeutic efficacy of the BA/KM combination, so as to make it more in line with clinical or practical application scenarios. Second, the risk of MRSA resistance induction caused by long-term use of this combination has not been explored yet. In the subsequent research, supplementary adaptive resistance experiments can be conducted to further verify its reliability in resistance control and enhance the persuasiveness of the conclusions. Third, although the lettuce model has initially demonstrated the application potential of this combination, in-depth analysis has not been carried out on the feasibility of this strategy in industrial large-scale production, the stability of the formulation during storage, and its compliance with regulatory requirements in food application scenarios. These factors are all key to affecting its practical transformation and require focused attention in future studies. Fourth, the interaction between BA and PBP2a/NorA proteins has so far only been predicted through molecular docking. In the future, experimental verification using techniques such as surface plasmon resonance, isothermal titration calorimetry, and gene mutagenesis will further improve the scientificity and credibility of the mechanism research.

## Figures and Tables

**Table 1:** Minimum inhibitory concentration (MIC) and fractional inhibitory concentration index (FICI) of baicalin and kanamycin against methicillin-resistant *Staphylococcus aureus* (MRSA) USA300 and *Staphylococcus aureus* ATCC29213.

Strains	Agents	MIC (µg/mL)		FICI	Outcome
		Alone	Combination		
MRSA USA300	baicalin kanamycin	1250 7.5	312.5 1.875	0.375	synergy
*Staphylococcus aureus* ATCC29213	baicalin kanamycin	2500 1.875	312.5 0.9375	0.625	partial synergy

Note: An FICI <0.5 indicates synergism, 0.5–0.75 indicates partial synergism, 0.75–1 indicates an additive effect, 1–4 indicates no effect, and >4 indicates antagonism.

## Data Availability

Data is contained in the paper.

## Abbreviations

AKP	Alkaline phosphatase
BA	Baicalin
CLSM	Confocal laser scanning microscopy
CR	Congo red
CV	Crystal violet
EDTA	Ethylene Diamine Tetraacetic Acid
EPS	Extracellular polymeric substance
FIC	Fractional inhibitory concentration
GM	Gentamicin
HADA	7-hydroxycoumarincarbonylamino-D-alanine
KM	Kanamycin
MIC	Minimum inhibitory concentration
MRSA	Methicillin-resistant *Staphylococcus aureus*
MTT	3-[4,5-dimethylthiazol-2-yl]-2,5-diphenyltetrazolium bromide
PBS	Phosphate-buffered saline
PIA	Polysaccharide intercellular adhesion
ROS	Reactive oxygen species
SD	Standard deviation
TSA	Tryptic soya agar
TSB	Tryptic soy broth
